# Increases in HIV Testing among Men Who Have Sex with Men — National HIV Behavioral Surveillance System, 20 U.S. Metropolitan Statistical Areas, 2008 and 2011

**DOI:** 10.1371/journal.pone.0104162

**Published:** 2014-09-02

**Authors:** Laura A. Cooley, Alexandra M. Oster, Charles E. Rose, Cyprian Wejnert, Binh C. Le, Gabriela Paz-Bailey, for the NHBS Study Group

**Affiliations:** Division of HIV/AIDS Prevention, National Center for HIV, Viral Hepatitis, STD and TB Prevention, Centers for Disease Control and Prevention, Atlanta, Georgia, United States of America; Public Health Agency of Barcelona, Spain

## Abstract

In 2011, 62% of estimated new HIV diagnoses in the United States were attributed to male-to-male sexual contact (men who have sex with men, MSM); 39% of these MSM were black or African American. HIV testing, recommended at least annually by CDC for sexually active MSM, is an essential first step in HIV care and treatment for HIV-positive individuals. A variety of HIV testing initiatives, designed to reach populations disproportionately affected by HIV, have been developed at both national and local levels. We assessed changes in HIV testing behavior among MSM participating in the National HIV Behavioral Surveillance System in 2008 and 2011. We compared the percentages tested in the previous 12 months in 2008 and 2011, overall and by race/ethnicity and age group. In unadjusted analyses, recent HIV testing increased from 63% in 2008 to 67% in 2011 overall (*P*<0.001), from 63% to 71% among black MSM (*P*<0.001), and from 63% to 75% among MSM of other/multiple races (*P*<0.001); testing did not increase significantly for white or Hispanic/Latino MSM. Multivariable model results indicated an overall increase in recent HIV testing (adjusted prevalence ratio [aPR] = 1.07, *P*<0.001). Increases were largest for black MSM (aPR = 1.12, *P*<0.001) and MSM of other/multiple races (aPR = 1.20, *P*<0.001). Among MSM aged 18–19 years, recent HIV testing was shown to increase significantly among black MSM (aPR = 1.20, *P* = 0.007), but not among MSM of other racial/ethnic groups. Increases in recent HIV testing among populations most affected by HIV are encouraging, but despite these increases, improved testing coverage is needed to meet CDC recommendations.

## Introduction

Approximately 1.1 million people in the United States are living with HIV [1], and an estimated 50,000 new infections occur each year [2]. Gay, bisexual, and other men who have sex with men (collectively referred to as MSM) and blacks or African Americans (hereafter referred to as blacks) continue to be disproportionately affected by HIV. While MSM represent only an estimated 4% of the male population in the United States [3], they accounted for approximately 79% of estimated new HIV diagnoses among adult and adolescent males and approximately 62% of estimated new HIV diagnoses overall in the United States in 2011 [4]. From 2008 to 2011, adult and adolescent MSM were the only group among whom the annual number of new HIV diagnoses increased [4]. Despite representing approximately 13% of the U.S. population [5], blacks accounted for 47% of estimated new HIV diagnoses in 2011 [4]. Considering the disproportionate burden of HIV among MSM and blacks, it is not surprising that the burden upon black MSM is also disproportionate; 39% percent of estimated new HIV diagnoses among MSM in 2011 occurred among black MSM [4]. Young black MSM are particularly affected. Though black MSM accounted for 39% of estimated new HIV diagnoses among MSM of all age groups in 2011, an estimated 58% of MSM aged 13–24 years diagnosed with HIV infection in 2011 were black [6].

Achieving awareness of HIV infection through testing is an essential first step to linking HIV-positive individuals to medical care and services, such as antiretroviral therapy, which can result in improved clinical outcomes [7]. Moreover, research has shown that once diagnosed with HIV, individuals reduce risk behaviors, which, in combination with viral suppression, can reduce the likelihood of transmitting HIV to others [8]–[10]. A recent analysis of U.S. HIV surveillance data estimated that of the adolescents and adults living with HIV infection at the end of 2010, 16% were living with undiagnosed infection [1]. Both the U.S. Department of Health and Human Services' *Healthy People 2020* and the National HIV/AIDS Strategy for the United States (NHAS) aim to reduce the percentage of persons living with undiagnosed HIV infection in the United States to 10% [11], [12].

CDC currently recommends that individuals at increased risk for HIV infection, including sexually active MSM, should undergo HIV testing at least annually [13]. According to CDC's Sexually Transmitted Diseases (STDs) Treatment Guidelines, 2010, testing for STDs, including HIV, among MSM is indicated even more frequently (every three to six months) under certain circumstances, such as for MSM with multiple or anonymous partners, MSM having sex in conjunction with illicit drug use, or MSM whose sex partners participate in these activities [14]. Previous analyses suggest that more frequent HIV testing may be beneficial for all MSM, irrespective of self-reported risk behavior [15], [16].

The importance of expanding HIV testing in populations disproportionately affected by HIV is described in NHAS [12]. Even before the release of NHAS in 2010, a variety of targeted testing initiatives were being developed at both national and local levels. CDC launched the Expanded HIV Testing Initiative (ETI) in 2007 to facilitate HIV diagnosis and linkage to medical care, focusing on the non-Hispanic black population. ETI was a three-year program intended to increase HIV testing in 25 U.S. jurisdictions; jurisdictions that received ETI funding were required to focus at least 80% of their activities on promoting opt-out HIV screening in high-morbidity clinical settings. With the remainder, they could support innovative methods to increase targeted HIV testing among high-risk populations (e.g., social networking approaches). For all persons newly diagnosed with HIV, jurisdictions had to ensure receipt of test results, linkage to medical care, and referral for partner services [17]. Jurisdictions were awarded between $510,000 and $6 million per year (for a total of $111 million), based proportionately on estimated 2005 AIDS diagnoses among blacks [18]. During 2007–2010, nearly 2.8 million tests were provided and more than 18,000 individuals were newly diagnosed with HIV. Sixty percent of these tests and 70% of the new diagnoses occurred among blacks [17]. A second ETI was funded in 2010 to expand routine testing services to reach a broader array of at-risk populations, including MSM (regardless of race or ethnicity) in 30 U.S. jurisdictions. A third initiative, intended to sustain progress made under the other two initiatives, was launched in 2012, extending eligibility to six additional jurisdictions [19].

Other testing initiatives arose during this time period, focused on populations likely overlapping with those targeted by CDC's ETIs. The Substance Abuse and Mental Health Services Administration (SAMHSA), for example, has funded multiple testing initiatives, including the Rapid HIV Testing Initiative (RHTI) during 2004–2007, designed to reduce HIV incidence among minority populations who may be at an even greater risk for acquiring or transmitting HIV associated with substance abuse and/or a mental health disorder [20]. In the private sector, the pharmaceutical company Gilead launched the HIV FOCUS program in 2010, to reduce the number of undiagnosed individuals through routine HIV screening in 10 cities heavily affected by HIV [21]. This list of initiatives, though far from an exhaustive review, helps illustrate the growing support for targeted testing throughout the public health community in the United States.

To describe HIV testing among MSM in the United States, we analyzed data from the National HIV Behavioral Surveillance System (NHBS). NHBS is a recurring cross-sectional survey designed to monitor selected sexual risk and drug-use behaviors, HIV testing experiences, use of prevention services, and HIV seroprevalence in three populations at high risk for HIV infection: MSM, injecting drug users, and heterosexual adults at increased risk of HIV infection [22]. Although previous analyses have examined differences in testing among various subpopulations of MSM [15], [16], [23], [24], the purpose of this analysis was to understand changes in HIV testing over time. Accordingly, the objectives of this analysis were to describe recent HIV testing (during the 12 months before interview) among MSM in 20 U.S. metropolitan statistical areas (MSAs) in 2008 and 2011, by race/ethnicity and age group, and then to determine if recent HIV testing changed from 2008 to 2011 among MSM, overall and by race/ethnicity and age group.

## Methods

### National HIV Behavioral Surveillance System

NHBS data are collected in three-year rounds of annual rotating cycles which focus on one population per year; methods for NHBS have been described in detail elsewhere [22], [23]. This analysis uses data from the MSM cycles of the second and third rounds of NHBS, conducted in 2008 and 2011 respectively. NHBS was conducted in 21 MSAs in 2008, and in 20 MSAs in 2011; the 20 MSAs common to both rounds were used in this analysis (Atlanta–Sandy Springs–Marietta, GA; Baltimore–Towson, MD; Boston–Cambridge–Quincy, MA–NH: Boston Quincy Division; Chicago–Joliet–Napierville, IL: Chicago–Joliet–Napierville Division; Dallas–Fort Worth–Arlington, TX: Dallas–Plano–Irving Division; Denver–Aurora–Broomfield, CO; Detroit–Warren–Livonia, MI: Detroit–Livonia–Dearborn Division; Houston–Sugar Land–Baytown, TX; Los Angeles–Long Beach–Santa Ana, CA: Los Angeles–Long Beach–Glendale Division; Miami–Ft. Lauderdale– Pompano Beach, FL: Miami Division; New Orleans– Metairie–Kenner, LA; New York–Northern New Jersey–Long Island, NY–NJ–PA: New York–White Plains–Wayne Division; New York–Northern New Jersey–Long Island, NY–NJ–PA: Nassau–Suffolk Division; New York–Northern New Jersey–Long Island, NY–NJ–PA: Newark–Union Division; Philadelphia–Camden– Wilmington, PA–NJ–DE–MD: Philadelphia Division; San Diego–Carlsbad–San Marcos, CA; San Francisco–Oakland– Fremont, CA: San Francisco–San Mateo–Redwood City Division; San Juan–Caguas–Guaynabo, PR; Seattle–Tacoma–Bellevue, WA: Seattle–Bellevue–Everett Division; and Washington–Arlington– Alexandria, DC–VA–MD–WV: Washington–Arlington–Alexandria Division). Together, these MSAs represented approximately 59% of the persons living with diagnosed HIV infection in large urban areas in 2010 [25].

### Sampling, Eligibility, and Data Collection

NHBS staff recruited MSM using venue-based (or time-space) sampling (VBS). Because there is no accepted sampling frame for a hidden population like MSM, standard probability-based sampling methods are not possible [26], [27]. VBS overcomes this limitation by sampling MSM where they congregate in the highest density, at known venues associated with the population [28]. Prior to recruitment, formative research was performed to identify appropriate venues, such as bars, clubs, social organizations, and street locations, that could be used for recruitment. In 2008, only venues at which 75% of men attending were MSM were eligible for inclusion. By 2011, venues had become more integrated by sexual orientation, thus this threshold was lowered to 50%. For each venue, days and times when MSM frequented these venues were also identified. From this list, venues and corresponding day/time periods were randomly selected for recruitment.

During recruitment, NHBS staff members systematically approached men for participation. For eligible men, informed consent was obtained, after which trained interviewers used a handheld computer to administer an anonymous, face-to-face interview. The standardized questionnaire, which lasted approximately 30 minutes, included questions regarding demographics, HIV-associated behaviors (including HIV testing history), and use of prevention and testing services. All participants, regardless of self-reported HIV infection status, were offered optional HIV testing, which was conducted after the interview in a private space. Participants received incentives for completing the interview and providing a specimen for HIV testing; the value varied by site, but $25 for the interview and an additional $25 for testing was typical.

Although our analysis criteria for both cycles were equivalent, eligibility criteria differed slightly for NHBS in 2008 versus 2011. For both surveys, individuals were considered eligible if they were born male and identified as male, were 18 years or older, had not previously completed any part of the current NHBS-MSM survey, resided in a participating MSA, were able to complete the survey in English or Spanish, and were capable of providing consent. In 2011, another requirement, reporting ever having had oral or anal sex with a man, was added [23].

### Ethics Statement

Activities for NHBS were approved by local institutional review boards (IRBs) for each of the 20 participating MSAs. The project underwent a CDC review and approval process; because CDC staff were determined not to be directly engaged for the purposes of NHBS research, CDC IRB approval was not required [29], [30]. All participants were explicitly assured during the recruitment process of the anonymous nature of the survey and the HIV testing. No personal identifiers were collected during enrollment, interview, or testing. All participants provided verbal informed consent to take part in the interview and to be tested for HIV. Verbal consent was documented electronically on the survey instrument by interviewers for all participants and on hard copy as required by local IRBs. Because data collection was anonymous, written consent was not possible and participant names or other personal identifiers were not linked to any NHBS instruments. All consent procedures, including verbal consent, were approved by local IRBs and CDC (see Table S1 for specific IRB information).

### Analysis Inclusion Criteria

We restricted our analysis to men from the 20 MSAs that participated in both 2008 and 2011 who reported at least one male sex partner in the 12 months before interview and who provided answers to the questions regarding history of HIV testing. Of men who reported a positive HIV test result, we included only those who reported that their first positive test result occurred during the 12 months before interview.

### Statistical Analysis

For 2008 and 2011, we compared the percentages of MSM who reported having a recent HIV test, stratified by race/ethnicity and age group. Next, we determined the percentages tested among each racial/ethnic group by age group. Bivariate analysis was performed using the chi-square test. All analyses were performed in SAS 9.3 (SAS Institute, Inc., Cary, NC).

Due to small sample sizes, we combined American Indian/Alaska Native, Asian, Native Hawaiian/other Pacific Islander, and multiple-race participants into one category for further analyses. Multiple-race participants accounted for less than 4% of the total sample both years. We conducted a hierarchical sensitivity analysis to assess the impact of testing among multiple-race blacks on single-race blacks and among multiple-race whites on single-race whites [31]. Specifically, we reclassified all multiple race blacks (including those who were biracial black/white) with the single-race blacks, and then, of the men who were left, we reclassified multiple race whites with the single-race whites. We reran the testing analysis and the results were unchanged. Because the results were similar either way, we left multiple-race blacks and whites with MSM of other/multiple races to be consistent with other NHBS publications.

To determine whether interview year was associated with recent HIV testing, we performed a multivariable analysis. Adjusted prevalence ratios (aPRs), *P*-values, and 95% confidence intervals (95% CIs) were estimated using a Poisson model with a robust standard error via the GENMOD procedure in SAS 9.3 [32]. Our main outcome was recent HIV testing (in the previous 12 months). To account for changes in the demographic composition of the sample and because demographic variables can be associated with HIV testing, the model was adjusted for race/ethnicity, age group, education, income, and MSA. From this model, we estimated the overall increase in recent HIV testing from 2008 to 2011. To explore whether temporal changes in recent HIV testing varied by race/ethnicity or age group, we then added interaction terms for race/ethnicity by interview year and age group by interview year. Next, to understand HIV testing among young MSM when stratified by race/ethnicity, we further expanded our model by adding a three-way interaction of race/ethnicity by age group by interview year, still using the overall sample.

Using the chi-square test, we examined the self-reported number of HIV tests in a two-year period to measure HIV testing frequency for men interviewed in 2008 and 2011. The percentage of men who tested twice in a two-year period was used as a proxy for annual testing; the percentage of men who were tested 3 or more times in two years was used to indicate testing more frequently than annually.

## Results

Our analysis included 16,069 MSM (7,943 [49%] from 2008 and 8,126 [51%] from 2011), which includes 298 MSM (145 from 2008 and 153 from 2011) who reported a first positive test result less than 12 months before interview. Response rates for men approached to participate in NHBS in 2008 and 2011 have been described previously [23]. Sample characteristics for the men included in this analysis are presented by year in Table 1. In both years, the largest percentage of the sample was non-Hispanic white (42% in 2008 and 39% in 2011), followed by Hispanic/Latino (26% in 2008 and 27% in 2011), and then non-Hispanic black (24% in 2008 and 26% in 2011). The median age of the sample was 31 years in 2008 and 30 years in 2011.

**Table 1:** Sample characteristics by year among men who have sex with men — National HIV Behavioral Surveillance System, 20 U.S. cities, 2008 and 2011.

	2008		2011	
	No.	(%)	No.	(%)
**Total**	7943		8126	
**Race/Ethnicity** *				
American Indian/Alaska Native	42	(1)	63	(1)
Asian/Native Hawaiian/Other Pacific Islanders	257	(3)	255	(3)
Black/African American	1926	(24)	2130	(26)
Hispanic/Latino	2037	(26)	2171	(27)
White	3361	(42)	3198	(39)
Multiple races	314	(4)	289	(4)
**Age group (years)**				
18–19	445	(6)	357	(4)
20–24	1509	(19)	1894	(23)
25–29	1561	(20)	1612	(20)
30–39	2194	(28)	1901	(23)
≥40	2234	(28)	2362	(29)
**Education**				
< High School	509	(6)	434	(5)
High School diploma or GED	1839	(23)	1961	(24)
Some college or technical degree	2537	(32)	2729	(34)
College degree or postgraduate education	3057	(38)	3002	(37)
**Annual Household Income**				
≤$19,999	2288	(29)	2484	(31)
$20,000–$39,000	2018	(25)	1983	(24)
$40,000–$74,999	1949	(25)	1958	(24)
≥$75,000	1563	(20)	1565	(19)

Percentages may not add to 100, due to rounding or missing values.

*Hispanics/Latinos can be of any race; categories are mutually exclusive.

Unadjusted prevalence of recent HIV testing in 2008 and 2011, as shown in Table 2, demonstrates an increase from 63% in 2008 to 67% in 2011 overall (*P*<0.001), from 63% to 71% among black MSM (*P*<0.001), and from 63% to 75% (*P*<0.001) among MSM of other/multiple races. Increases were not statistically significant for Hispanic/Latino MSM (from 63% to 65%, *P* = 0.2) or white MSM (from 64% to 66%, *P* = 0.2). Multivariable analyses demonstrate an overall adjusted increase in recent HIV testing (aPR = 1.07, *P*<0.001). This increase varied significantly by race/ethnicity (*P*<0.001 for the interaction term). Consistent with the unadjusted results, the adjusted increases in testing were significant for black MSM (aPR = 1.12, *P*<0.001) and MSM of other/multiple races (aPR = 1.20, *P*<0.001) (Table 2).

**Table 2:** HIV testing in previous 12 months among men who have sex with men — National HIV Behavioral Surveillance System, 20 U.S. cities, 2008 and 2011.

	2008		2011					
	No. Tested	(%)	No. Tested	(%)	*P*-value*	aPR	(95% CI)	*P*-value†
						2011 vs 2008		
**Interview year** §	5026	(63)	5478	(67)	**<0.001**	**1.07**	**(1.05,1.09)**	**<0.001**
**Race/Ethnicity** ** **by interview year** ¶								
Black/African American	1213	(63)	1510	(71)	**<0.001**	**1.12**	**(1.07,1.17)**	**<0.001**
Hispanic/Latino	1275	(63)	1402	(65)	0.2	1.02	(0.98,1.08)	0.3
White	2146	(64)	2096	(66)	0.2	1.03	(0.98,1.07)	0.2
Other/multiple races	387	(63)	456	(75)	**<0.001**	**1.20**	**(1.11,1.30)**	**<0.001**
**Age group by interview year** ¶								
18–19	288	(65)	238	(67)	0.6	1.05	(0.95,1.17)	0.3
20–24	1045	(69)	1364	(72)	0.08	**1.06**	**(1.02,1.11)**	**0.008**
25–29	1066	(68)	1180	(73)	**0.002**	**1.10**	**(1.05,1.15)**	**<0.001**
30–39	1425	(65)	1322	(70)	**0.002**	**1.10**	**(1.05,1.15)**	**<0.001**
≥40	1202	(54)	1374	(58)	**0.003**	**1.14**	**(1.08,1.20)**	**<0.001**

aPR  =  Adjusted Prevalence Ratio and CI  =  Confidence Interval.

*Unadjusted *P*-value.

† Adjusted *P*-value.

§ Adjusted for race/ethnicity, age group, education, income, and MSA; aPR presented for interview year.

¶ Adjusted for race/ethnicity, age group, education, income, MSA, race/ethnicity by interview year, and age group by interview year; aPR presented for the interaction terms, race/ethnicity by interview year and age group by interview year.

**Hispanics/Latinos can be of any race; categories are mutually exclusive.

The increase in recent testing did not vary overall by age group (*P* = 0.4 for the interaction term). Nevertheless, knowing that young MSM, and particularly young black MSM, are disproportionately affected by HIV, we investigated further, using the three-way interaction of race/ethnicity by age group by interview year to clarify differences in recent HIV testing by race/ethnicity among the youngest MSM. Although adjusted increases in recent HIV testing (Table 2) were not significant for young MSM aged 18–19 years (aPR = 1.05, *P* = 0.3), black MSM aged 18–19 years experienced significant increases in recent HIV testing (aPR = 1.20, *P* = 0.007), while MSM aged 18–19 years of other racial/ethnic groups did not (Table 3).

**Table 3:** HIV testing in previous 12 months, stratified by race/ethnicity and age group, among men who have sex with men — National HIV Behavioral Surveillance System, 20 U.S. cities, 2008 and 2011.

	2008		2011					
	No. Tested	(%)	No. Tested	(%)	*P*-value*	aPR†	(95% CI)	*P*-value†
						2011 vs 2008		
**Race/Ethnicity** § **by age group by interview year**								
**Black/African American**								
18–19	137	(65)	129	(77)	**0.009**	**1.20**	**(1.05,1.38)**	**0.007**
20–24	361	(71)	508	(76)	**0.03**	1.07	(0.999,1.15)	0.055
25–29	247	(66)	323	(75)	**0.006**	**1.12**	**(1.03,1.23)**	**0.01**
30–39	282	(62)	296	(73)	**<0.001**	**1.16**	**(1.06,1.27)**	**0.002**
≥40	186	(49)	254	(55)	0.1	1.12	(0.98,1.27)	0.09
**Hispanic/Latino**								
18–19	95	(66)	72	(55)	0.06	0.87	(0.72,1.06)	0.2
20–24	277	(64)	401	(68)	0.1	1.08	(0.98,1.18)	0.1
25–29	284	(66)	302	(67)	0.9	0.98	(0.89,1.07)	0.7
30–39	389	(66)	376	(69)	0.4	1.04	(0.96,1.13)	0.3
≥40	230	(52)	251	(56)	0.2	1.08	(0.96,1.22)	0.2
**White**								
18–19	39	(63)	23	(58)	0.6	0.87	(0.62,1.21)	0.4
20–24	312	(71)	328	(69)	0.5	0.98	(0.90,1.07)	0.7
25–29	429	(72)	432	(74)	0.3	1.05	(0.98,1.12)	0.2
30–39	642	(66)	531	(68)	0.4	1.03	(0.97,1.10)	0.3
≥40	724	(56)	782	(59)	0.1	**1.07**	**(1.003,1.14)**	**0.04**
**Other/multiple races**								
18–19	17	(61)	13	(72)	0.4	1.21	(0.78,1.88)	0.4
20–24	93	(75)	125	(78)	0.5	1.04	(0.91,1.19)	0.5
25–29	105	(65)	119	(83)	**<0.001**	**1.32**	**(1.15,1.51)**	**<0.001**
30–39	111	(63)	113	(70)	0.1	1.12	(0.96,1.30)	0.1
≥40	61	(50)	86	(69)	**0.002**	**1.44**	**(1.17,1.79)**	**<0.001**

aPR  =  Adjusted Prevalence Ratio and CI  =  Confidence Interval.

*Unadjusted *P*-value.

† Adjusted for race/ethnicity, age group, education, income, MSA, race/ethnicity by interview year, age group by interview year, race/ethnicity by age group, and race/ethnicity by age group by interview year; aPR presented for the three-way interaction term, race/ethnicity by age group by interview year.

§ Hispanics/Latinos can be of any race; categories are mutually exclusive.

**Figure 1:**
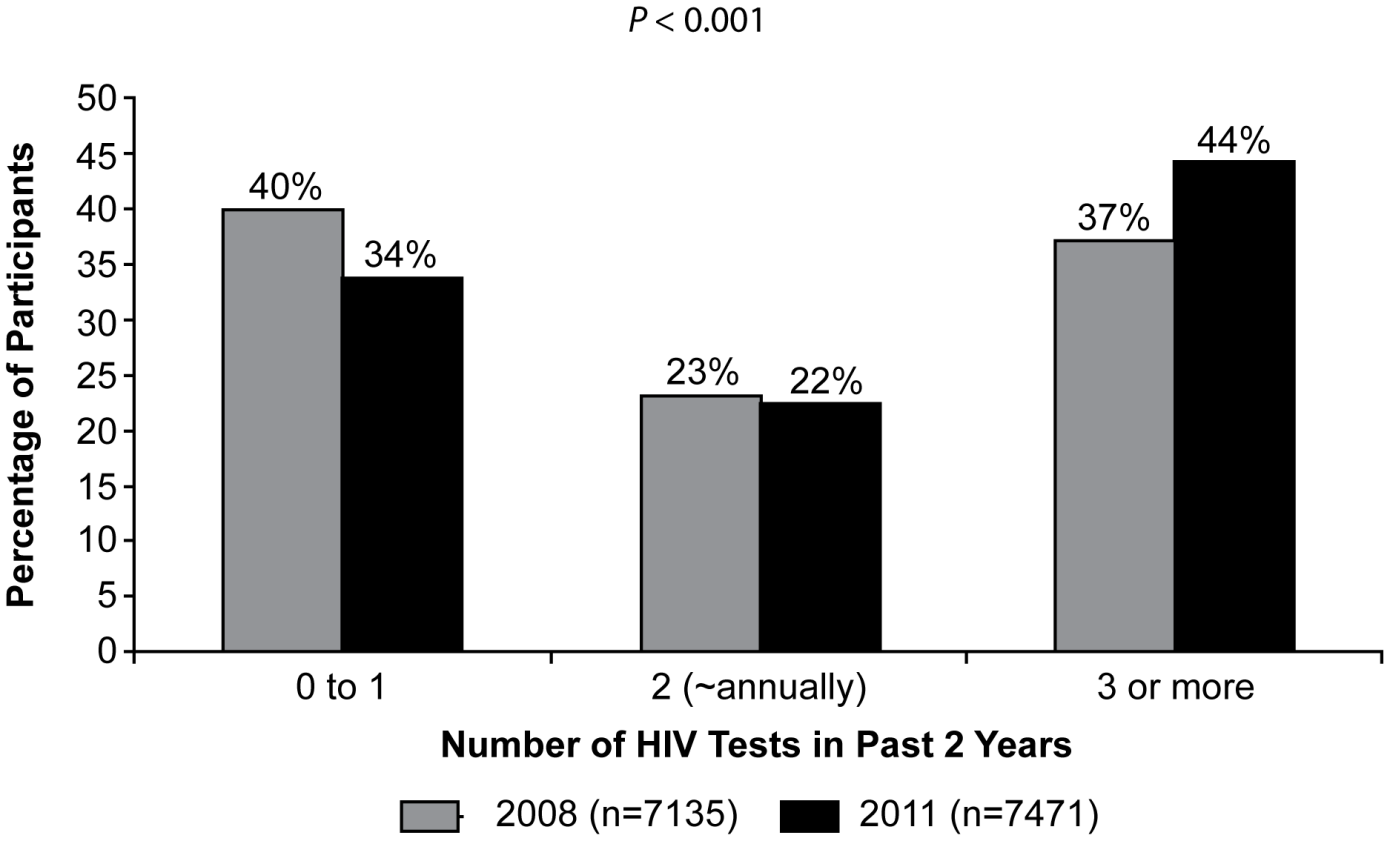
Number of HIV tests in a two-year period among MSM in 2008 and 2011. During the interview, NHBS participants were asked to report their HIV testing frequency in the two years before interview. Testing twice in a two-year period was used as a proxy for annual testing. The distribution of HIV testing frequency among MSM during the 2 years before interview, shown here, changed from 2008 to 2011 (P<0.001). The percentage of men who tested one time or less in the 2 years before interview (less than annually) decreased from 40% in 2008 to 34% in 2011. The percentage who tested twice in two years (annually) was similar (23% to 22%), while the percentage of men who tested three or more times in two years (more than annually) increased from 37% to 44%.

As shown in Figure 1, the distribution of HIV testing frequency among MSM during the 2 years before interview changed from 2008 to 2011 (P<0.001). The percentage of men who tested one time or less in the 2 years before interview (less than annually) decreased from 40% in 2008 to 34% in 2011. The percentage who tested twice in two years (annually) was similar (23% to 22%), while the percentage of men who tested three or more times in two years (more than annually) increased from 37% to 44%.

## Discussion

In this analysis of NHBS data from 20 U.S. MSAs, we found that recent HIV testing increased among MSM from 2008 to 2011. Increases were more substantial among certain racial/ethnic groups, specifically black MSM. Increases were also substantial for MSM of other/multiple races, however, these men accounted for a small percentage of the total sample. Although we did not see significant increases in recent HIV testing among the youngest MSM overall, we did see significant increases among young black MSM, a subpopulation particularly affected by HIV. These differential increases might reflect an effect of testing initiatives focused on populations disproportionately affected by HIV.

Even in 2011, only 67% of the MSM participating in NHBS had been tested for HIV during the previous 12 months. According to NHBS data from 2008, the main reasons for not undergoing recent HIV testing included a perception of being at low risk for HIV infection, followed by a fear of testing positive, followed by a lack of time [22]; results for 2011 were similar (unpublished NHBS data). Data from another survey suggest that recent HIV testing may be even lower in different populations, for instance, MSM from smaller cities or rural areas. The National Survey of Family Growth (NSFG) estimates that only 39% of sexually active MSM interviewed during 2006–2010 had been tested for HIV in the previous 12 months [33]. Although differences in the geographic areas, sampling methods, and mode of interview administration between NSFG and NHBS limit the comparability of the results, the basic message is the same; measures of recent HIV testing among MSM suggest that CDC recommendations for HIV testing frequency among MSM—that all sexually active MSM undergo HIV testing at least annually—have not yet been met. Although increases in recent HIV testing were observed in our analysis, there is more work to be done to increase HIV testing among populations most affected by HIV. HIV testing continues to play a critical role in the fight against HIV [7]–[10], and additional HIV testing efforts might benefit the MSM population.

Of MSM participating in NHBS who had tested for HIV in the previous 12 months, 5% were HIV-positive but unaware of their infection in 2011 [16], while 7% were in 2008 [15]. Furthermore, of the participants in both 2008 and 2011 who were found to be HIV-positive but unaware of their infection, approximately one third had been tested for HIV during the past six months [15], [16]. These data suggest that, at least among sexually active MSM, annual testing may not be sufficient. In populations with a high HIV burden, more frequent HIV testing may be indicated. In our analysis, we found that the percentage of MSM who tested at least three times during the two years before interview increased, perhaps an early sign that at least some MSM are undergoing HIV testing more frequently.

A recently published analysis of 2008 and 2011 NHBS data showed that HIV prevalence among MSM participating in NHBS was stable from 2008 to 2011, however, awareness of infection among HIV-positive MSM increased during that time period [23]. Increased testing, among other factors such as reduced HIV stigma, may be contributing to the increase in awareness of HIV infection [34]. HIV surveillance data show that the annual number of HIV diagnoses among MSM continues to increase [4]. Increased testing, in addition to other factors such as increased incidence [2], may help explain this increase in diagnoses. Additional analyses are needed to examine this hypothesis and relationship.

This analysis is subject to several limitations. First, because the data were collected in 20 large MSAs with high HIV burden and most men were recruited from bars or dance clubs, study findings may be generalizable only to MSM who attend venues in large urban areas. Recruiting participants at venues, as is done with VBS, may result in selection bias; these data are not weighted to account for bias. Furthermore, the venue inclusion criteria changed from 2008 to 2011. Next, because date of most recent HIV test was self-reported, social desirability and recall biases may affect estimates. Finally, this study is cross-sectional and cannot be linked to any particular testing initiative, including CDC's ETI, thus causality cannot be established. Seventeen of the 20 NHBS sites were among the original 25 ETI jurisdictions, but despite the considerable overlap, NHBS was not designed to evaluate ETI.

### Conclusions

As theorized in national strategic initiatives, achieving increased awareness of HIV infection through HIV testing is an important step towards reducing new HIV infections by leading to linkage to and engagement in HIV care, viral suppression, and behavioral change [7]–[10]. We found that recent HIV testing increased from 2008 to 2011 among black MSM, including young black MSM, which are populations disproportionately affected by HIV. HIV testing initiatives focused on populations most affected by HIV might be having a positive effect. While the finding of increased HIV testing among certain populations is encouraging, our analysis demonstrates that improved coverage of HIV testing is needed to meet CDC recommendations.

## Supporting Information

Table S1 **National HIV Behavioral Surveillance System (NHBS): Local institutional review boards (IRBs) by Metropolitan Statistical Area (MSA).**
(DOCX)
