# Supplementary material for: Increases in HIV Testing among Men Who Have Sex with Men — National HIV Behavioral Surveillance System, 20 U.S. Metropolitan Statistical Areas, 2008 and 2011
Source: PLoS One. 2014 Sep 2;9(9):e104162. doi: 10.1371/journal.pone.0104162 (PMC4151966; doi:10.1371/journal.pone.0104162)
Supplement: Table S1 — National HIV Behavioral Surveillance System (NHBS): Local institutional review boards (IRBs) by Metropolitan Statistical Area (MSA). (DOCX) [file pone.0104162.s001.docx]

| **MSA** | **Approving IRB for NHBS-MSM activities (2008 and 2011)** |
| --- | --- |
| ATLANTA | Georgia Department of Human Resources IRB (2008 only)  Emory University IRB (2008 only)  Georgia Department of Public Health (2011 only)  Georgia Department of Community Health (2011 only) |
| BALTIMORE | Maryland Department of Health and Mental Hygiene IRB  Johns Hopkins Bloomberg School of Public Health IRB |
| BOSTON | Massachusetts Department of Public Health Human Research Review Committee  Boston University Medical Center IRB (2011 only) |
| CHICAGO | Chicago Department of Public Health IRB |
| DALLAS | Texas Department of State Health Services IRB  Texas A&M University Office of Research Compliance |
| DENVER | Colorado Multiple IRB |
| DETROIT | Michigan Department of Community Health IRB |
| HOUSTON | Univ. of Texas Health Science Center Committee for the Protection of Human Subjects |
| LA | County of Los Angeles Public Health IRB |
| MIAMI | Florida Department of Health IRB  University of Miami IRB (2008 only) |
| NASSAU | State of New York Department of Health IRB |
| NEW ORLEANS | State of Louisiana Department of Health and Hospitals IRB  Louisiana State University Health and Sciences Center IRB |
| NYC | New York City Department of Health and Mental Hygiene IRB  National Development and Research Institute IRB (2008 only)  John Jay College of Criminal Justice IRB (2011 only) |
| NEWARK | State of New Jersey Department of Health and Senior Services IRB |
| PHILADELPHIA | City of Philadelphia Department of Public Health IRB |
| SAN FRANCISCO | Univ. of California, San Francisco Committee on Human Research |
| SAN JUAN | Univ. of Puerto Rico, Medical Sciences Campus IRB |
| SAN DIEGO | California Health and Human Services Agency Committee for the Protection of Human Subjects |
| SEATTLE | State of Washington Department of Social and Health Services IRB |
| DC | Government of the District of Columbia Department of Health IRB  George Washington University Medical Center IRB |

**National HIV Behavioral Surveillance System (NHBS): Local institutional review boards (IRBs) by Metropolitan Statistical Area (MSA)***

*CDC approval was obtained via CDC review and approval process ( 45 CFR 46 regulations)
